# Provenance Variation in Morphology, Nutrient Status and Total Phytochemical Contents of *Alpinia hainanensis* K. Schum. Under a Mixed Valuable-Tree-Species Forest

**DOI:** 10.3390/plants15111602

**Published:** 2026-05-23

**Authors:** Meng Liang, Anjia Huang, Xinyu Liu, Zhoujun Li, Tianbao Jiang, Daocheng Ma, Mei Yang

**Affiliations:** 1 Guangxi Colleges and Universities Key Laboratory for Cultivation and Utilization of Subtropical Forest Plantation, Guangxi University, Nanning 530004, China; liangmengyz@163.com (M.L.); huanganjia147@163.com (A.H.); liuxinyu@163.com (X.L.); lizhoujunmail@163.com (Z.L.); jtb051005@163.com (T.J.); 2 School of Forestry, Guangxi University, Nanning 530004, China

**Keywords:** *Alpinia hainanensis*, morphological characteristics, nutrient and chemical components accumulation, understory introduction performances, provenances selection

## Abstract

*Alpinia hainanensis* Hayata (family Zingiberaceae) is a medicinal plant with high potential for understory cultivation, but the variations in growth, morphological status, physiological conditions, nutrient absorption and phytochemical contents between different understory provenances originating from southern China remain poorly understood. In this study, different growth, physiological and phytochemical indices of three provenances [Hainan (HN) and Bobai (BB)/Baise (BS) from Guangxi] were determined, in order to better evaluate their introduction potentials. The results showed that two Guangxi provenances (BB and BS) showed superior aboveground growth and biomass accumulation compared to the HN provenance, yet most the leaf functional traits and medicinal qualities of some organs were similar. Sprout and leaf growth were consistent, and a negative correlation was observed between leaf nitrogen and flavonoid content. Based on principal component analysis (PCA), BB performed better than two other provenances in growth and phytochemical aspects. Due to the limited number of collections of provenances and absence of microclimate data, these findings should be considered preliminary. This study provides a basis for provenances selection and understory cultivation in *A. hainanensis* in (sub)tropical regions.

## 1. Introduction

“Forest-medicinal plant” agroforestry systems have been widely promoted in China, India, Indonesia and many other Asian, African and (sub)tropical regions, as they improve regional economy development and land utilization [1,2,3]. Proper intercropping of forest and medicinal plants is also beneficial to the understory environment [4]. At present, various medicinal plants are cultivated in different artificial or economic forests in China, including *Stemona japonica*, *Curcuma longa*, *Amomum villosum*, *Alpinia oxyphylla* and *Ficus hirta* and others, which could also achieve better growth status and medicinal quality [5,6,7]. However, the tolerance and phytochemical components showed significant differences between different provenances, varieties and organs among the same species [8,9]. Therefore, it is important to select suitable provenances of various medicinal plants for understory systems foundation.

The morphology, growth status, and inclusions contents showed significant differences between provenances, which could be divided into some main categories [10]. Some morphological indices would show close correlations between aboveground status and total phytochemical contents (including total polysaccharides, saponin contents, etc.), such as *Polygonatum cyrtonema*, *Crataegus* spp., *Falcaria vulgaris* and so on [11,12,13,14]. The anatomical structure and various physiological indices of *Mentha mozaffarianii*, *Aspidopterys concave* and many other species could also be identified and evaluated to reflect the adaptability and resistance of the plant to the external environment. They are also important criteria for evaluating the adaptability and promotion potential of germplasm resources [15,16]. For example, six *Aspidopterys concave* provenances could be classified into three categories based on leaf morphological and physiological indices. Daxin and Longzhou provenances from Guangxi Zhuang Autonomous Region are suitable for cultivation in subtropical regions in China and some other Asian countries [17]. Total phenols, flavonoids and other phytochemical contents of *Habenaria edgeworthii* vary significantly within and between provenances, and these traits could be used for best resource selection [18]. The differences in phytochemical contents and macronutrient contents could be used to select better provenances for promotion. In summary, morphological, physiological and nutrient/phytochemical characteristics play significant roles in provenance selection and introduction.

Zingiberaceae species show strong adaptability in soil and canopy conditions in tropical and subtropical regions, thereby inhabiting various understory environments [19,20,21]. So understory space is suitable for growth and provenance protection in Zingiberaceae species. *Alpinia hainanensis* K. Schum. is a kind of traditional phytomedicine found in many south Asian countries with high medicinal value. At present, studies on *A. hainanensis* are mainly about the medicinal quality changes during fruit development and maturation [22], the foundation of “Rubber-*A. hainanensis*” agroforestry systems [23], root exudate metabolites [24] and so on. Although fruits and seeds were the main medicinal organs, its vegetative organs always constitute dominant harvestable biomass, and they show different phytochemical contents between provenances [25,26]. So the phytochemical contents among different organs of *A. hainanensis* also need to be further investigated. Thus, Zingiberaceae plant species are widely cultivated in the understories of forests in China and tropical Asia. Appropriate selection and promotion should be carried out based on their ecological characteristics, growth performance in the forest understory and planting effects.

In summary, proper understory cultivation and provenance selection have become significant aspects of Zingiberaceae-based agroforestry systems. However, there are few studies about the comparisons between aboveground growth status, physiological adaptation, and phytochemical profiles of different southern China *A. hainanensis* provenances under mixed valuable-tree-species forest. We determined the growth conditions, morphological, physiological characteristics and nutrient/total phytochemical accumulation in various organs of different *A. hainanensis* provenances [Hainan (HN), Baise (BS) and Bobai (BB)] in this study. We hypothesized that (1) there were significant differences in different traits among the three provenances; (2) the provenances originating from Guangxi outperformed the others in growth and biomass accumulation due to better local adaptation; (3) there were close relationships between growth and phytochemical component accumulation, with slower-growing provenances accumulating higher levels of defensive compounds (e.g., flavonoids, phenols). The objectives were (1) to identify external morphological and phytochemical properties of different *A. hainanensis* provenances and (2) to select the best *A. hainanensis* provenance in order to establish proper “*A. hainanensis*-mixed forest” agroforestry systems. The findings of this study build a solid foundation for Zingiberaceae provenance selection and promotion in Guangxi and even other (sub)tropical regions in the world.

## 2. Materials and Methods

### 2.1. Test Site

The test site for *A. hainanensis* provenances was chosen under as valuable-tree-species forest (dominated by various Fagaceae, Magnoliaceae, Lauraceae native species, which were treated as precious large-diameter timber species) in the Arboretum of Guangxi Academy of Forestry Sciences (22°56′ N, 108°21′ E), with a subtropical climate of the average annual temperature of 20~21 °C, the annual precipitation of 1200~1300 mm, the altitude of 100~237 m, and the annual sunshine duration of 1450~1650 h. The average canopy densities of the upper forest were ranging from around 0.8 to 0.9, which was suitable for the growth of *A. hainanensis* seedlings. The main species of the upper forest layer were *Quercus variabilis*, *Michelia chapensis*, *Cryptocarya concinna* and *Cyclobalanopsis patelliformi*.

### 2.2. Experimental Design

**Figure 1:**
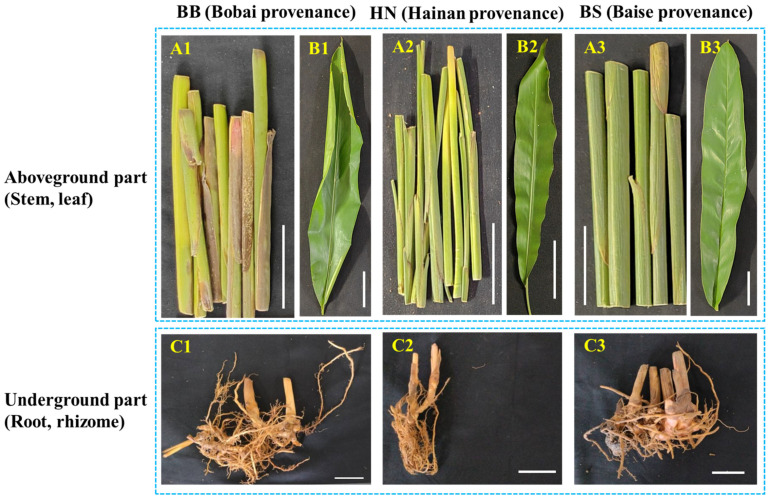
Morphological characteristics of the aboveground and underground parts of different *A. hainanensis* provenances. Note: (**A1**,**B1**,**C1**) represent the stems, leaves and the underground parts (roots and rhizomes) of Bobai (BB) provenance seedlings; (**A2**,**B2**,**C2**) represent the stems, leaves and the underground parts (roots and rhizomes) of Hainan (HN) provenance seedlings; (**A3**,**B3**,**C3**) represent stems, leaves and the underground parts (roots and rhizomes) of Baise (BS) provenance seedlings; the length of the white bars in this figure represents 5 cm.

In March 2022, seeds of three *A. hainanensis* provenances [(originated from Hainan (HN), Bobai (BB, Guangxi) and Baise (BS, Guangxi)] were provided by Guangxi Nanyao Co., Ltd., Yulin, China (which were collected from 3~4-year-old mother plants) and transplanted into plastic pots (with 20 cm diameter and 15 cm height) with the same mixed substrates (V_soil_: V_sand_ = 3:1. pH 4.50, total nitrogen 1.76 g/kg, total phosphorus 1.02 g/kg, total potassium 3.27 g/kg). After preliminary cultivation, they were transplanted under the mixed valuable-tree-species forest in November 2023. All three provenances were taxonomically identified by Dr. Rongyan Deng (School of Forestry, Guangxi University) based on morphological characteristics following the Flora of China. Voucher specimens were not available because the seedlings were commercially sourced. However, representative photographs of each provenance are shown in Figure 1. Planting holes (20 cm length, width and depth were dug for *A. hainanensis* plantation, and one seedling was planted in each hole with 500 g compound fertilizer at the bottom mixed with soil, which was provided by Guangxi Huawote Fertilization Co., Ltd. in Nanning, China, N:P_2_O_5_:K_2_O = 15:15:15). The planting positions of the three provenances were randomized with independent plots, and the micro-site variation was controlled. All of their planting spaces were 1.5 m × 2 m, and the planting density was 3333 plants per hectare. The pH and basic soil properties (0~20 cm, the depth of soil layer for growing *A. hainanensis* provenances cultivation) were pH 3.62, ammonium nitrogen (AN) 15.37 mg/kg, available phosphorus (AP) 10.21 mg/kg, and available potassium (AK) 40.36 mg/kg. The experiment field was weeded every half a year (with no more fertilizer additions), and the other management methods were the same between different provenances. The aboveground growth status and leaf morphological traits were determined in March 2024. In September 2024, the physiological characteristics, nutrient contents and chemical component contents were determined, and the best provenances among these three provenances were selected via these indices.

### 2.3. Determination of Indices

#### 2.3.1. Aboveground Growth Indices

Thirty *A. hainanensis* seedlings were randomly selected to determine aboveground growth indices in each provenance (including plant height, stem diameter at the ground level, sprouting numbers and leaf number). Plant height and stem diameter at the ground level were measured by a steel ruler and a digital vernier caliper (with the accuracy within 0.01 cm and 0.01 mm, respectively). The sprouting and leaf number of *A. hainanensis* seedlings were recorded by visual observation [27]. There were thirty biological replicates for these indices of each provenance.

#### 2.3.2. Leaf Morphological and Functional Traits

Three *A. hainanensis* seedlings were randomly selected in each provenance to determine the leaf morphological and functional traits (the 3rd~5th mature functional leaves counted from the top bud of the seedlings were selected, and the average of these three leaves was treated as the replicate of each index). As for leaf morphological traits, leaf length, the maximum leaf width, 1/2 leaf width and leaf stalk length were determined by steel ruler (with the accuracy of 0.01 cm); leaf thickness was determined by a digital vernier caliper (with the accuracy of 0.01 mm). After determination, the leaf shape index (LSI, the ratios of leaf length and the maximum leaf width), the ratios of maximum leaf width and 1/2 leaf width (WWR), and the ratios of leaf length and 1/2 leaf width (LWR) could be calculated. As for leaf functional traits, the single-leaf area was determined by handheld leaf area meter (CI-203, with the accuracy of 0.01 cm^2^). Single-leaf fresh weight was determined by electronic scales (with the accuracy of 0.01 g), of which the single-leaf dry weight was determined after 30 min (105 °C) sterilizing and 75 °C drying (until constant weight). Moisture content (% = the water content/fresh weight), specific leaf area (cm^2^/g = single-leaf area/single-leaf weight) and specific leaf weight (g/cm^2^ = single-leaf weight/single-leaf area) could be calculated [28]. There were three biological replicates for these indices for each provenance.

#### 2.3.3. Plant Chlorophyll and Soluble Sugar Contents

The mature functional leaves that were used to determine the physiological indices were the same as the fresh samples which were used to determine morphological traits. Leaf chlorophyll and soluble sugar contents were determined referring to Ma et al., 2024 [29]. There were three biological replicates for these indices of each provenance. Detailed methods were as follows:

1. Chlorophyll content: About 0.1~0.2 g of fresh leaves was cut into about 2~5 mm^2^ pieces and added to 5 mL mixed extraction solution (V_Acetone_:V_Absolute ethanol_:V_Deionized water_ = 4.5:4.5:1) and placed in the dark environment until the tissues completely turned white (nearly 48 h). The extracted supernatant was used for chlorophyll determination (under 663 and 645 nm wavelengths with an ultraviolet spectrophotometer (UV-2450; Shimadzu, Tokyo, Japan)). In the following formula, “V” represents the volume of extraction liquid (5 mL), and “W” represents fresh leaf sample weight.
  (1) Chl a = (12.70OD663 − 2.69OD645) × (V/1000 W)
  (2) Chl b = (22.90OD645 − 4.68OD663) × (V/1000 W)
  (3) Chl a + b = (20.20OD645 + 8.02OD663) × (V/1000 W)
2. Soluble sugar content: About 0.1~0.2 g of dried leaf sample (passed 60-mesh sieves) was added to 10~15 mL deionized water (with twice 0.5 h boiling water bath). The supernatants were combined into 50 mL volumetric flasks and fixed with deionized water. Then, 0.5 mL extraction and 0.5 mL anthrone ethyl acetate reagent (including 0.5 g anthrone + 100 mL ethyl acetate) were added into 50 mL test tubes; 5 mL concentrated sulfuric acid was added into the mixture and the tubes were placed into a 1 min boiling water bath. The mixture (turned blue) was cooled and used for soluble sugar determination (under 630 nm wavelengths) with the INFINITE M200 PRO instrument (Tecan, Männedorf, Switzerland).

#### 2.3.4. Plant Nutrient Content and Stoichiometric Ratios

The dried leaf samples (the same as the dried samples of leaf dry weight determination) of each provenance were ground and passed through 60-mesh sieves for nutrient content determination. H_2_SO_4_-H_2_O_2_ was used to digest the powder samples, and the supernatant (adjusted to 100 mL) was used to determine TN (Total Nitrogen), TP (Total Phosphorus) and TK (Total Potassium) contents (which were three biological replicates for each provenance using an AA3 continuous flow chemical analyzer (SEAL Analytical, Norderstedt, Germany), molybdenum-antimony anti-colorimetric method and flame photometer method, respectively) [30]. Meanwhile, the stoichiometric ratios of TN/TP, TN/TK, and TK/TP were calculated. There were three biological replicates for these indices of each provenance.

#### 2.3.5. Plant Total Phytochemical Contents

Dried samples of *A. hainanensis* seedlings (roots, rhizomes, stems and leaves) from three random plants each provenance were used to determine chemical component contents; 0.2 g dried samples were put in 15 mL plastic falcon tubes with 20 mL aliquot of 75% (*v*/*v*) ethanol (extracted at 60 °C for 30 min). After extraction, the liquid supernatants were preserved. AlCl_3_, the Folin phenol method (Folin–Ciocalteu assay) and the ninhydrin method were respectively used for flavonoid, phenol and amino acid content determination [31]. There were three biological replicates for these indices of each provenance. Detailed methods were as follows:

(1) Total flavonoid content: 1 mL extraction was added into test tubes; 0.3 mL 5% NaNO_2_ (aq), 0.3 mL 10% AlCl_3_ (aq), 4 mL 4% NaOH (aq) and 4.4 mL 75% ethanol (aq, *V*/*V*) were added (with 6 min stirring and mixing after adding each solution). The mixture (turned red) was used for total flavonoid content determination (under 510 nm wavelengths) with the INFINITE M200 PRO instrument (Tecan, Männedorf, Switzerland).
(2) Total phenol content: 1 mL extraction was added into test tubes; 1 mL Folin–Ciocalteu solution and 2 mL 12% Na_2_CO_3_ (aq) and 6 mL 75% ethanol (aq, *V*/*V*) was added. The mixture was put into the dark for 1 h and was used for total phenol content determination (under 765 nm wavelength) with the INFINITE M200 PRO instrument (Tecan, Männedorf, Switzerland).
(3) Total amino acid content: 1 mL extraction was added into test tubes; 4 mL CH_3_COONa-CH_3_COOH (aq, pH = 6.0) and 1 mL ninhydrin reagent were added in order. The mixture was used for a 92 °C boiling water bath and used for total amino acid content determination under 569 nm wavelength with the INFINITE M200 PRO instrument (Tecan, Männedorf, Switzerland).

### 2.4. Statistical Analysis

All the morphological, physiological and nutrient/total phytochemical content indices were organized and descriptive analyzed by Microsoft Excel 2016. According to the Shapiro–Wilk test and homogeneity of variance (Levene’s test) results, all the data met the assumptions for ANOVA. IBM SPSS 19.0 was used for one-way ANOVA analysis (α = 0.05 and α = 0.01), multiple comparisons (via Duncan’s test) and correlation analysis (via Pearson bivariate analysis, α = 0.05 and α = 0.01). Finally, WPS Office 2016 was used to make all the tables, figures and heatmaps in the text.

## 3. Results

### 3.1. Variations in Plant Phenotype

#### 3.1.1. Aboveground Growth and Morphological Differences

There were significant differences in morphological characteristics and aboveground growth status between the three *A. hainanensis* provenances. The seedlings of HN (Hainan) provenance had smaller leaves, underground parts and thinner stems, while BB (Bobai) and BS (Baise) provenances (from Guangxi) showed the opposite trend. Leaves of HN and BB provenances had slight wave-like shapes at the edge of leaves, while the leaves of BS provenance had smooth edges (Figure 1). There were also significant (*p* < 0.05, same below) or highly significant (*p* < 0.01, same below) differences in the four aboveground indices between the three provenances; seedlings of BS provenance had the highest plant height and stem diameter values at the ground level (73.79 cm and 6.63 mm), while BB provenance seedlings had most sprouts and leaves (2.67 and 18.57). The coefficient of variation (c.v.) in BS provenance had the highest values among the three provenances, ranging from 26.09% to 48.07% (much higher than the other two provenances), and the average coefficient of variation (c.v.) decreased in the following order: leaf number (34.23%) > sprouting number (33.26%) > stem diameter at the ground level (22.37%) > plant height (21.47%), indicating that the sources of variation among different provenances mainly come from the differences in sprout and leaf growth (Table 1 and Table S1). In summary, the growth conditions of the two provenances of *A. hainanensis* seedlings in Guangxi were superior to the HN provenance, and the coefficient of variation was also higher. The BS provenance was the most prominent.

#### 3.1.2. Leaf Morphological/Functional Characteristics

As for leaf morphological indices, there were highly significant differences between most of the indices except for maximum leaf width, 1/2 leaf width and LWR. Leaves of BB provenance had the highest leaf length (29.27 cm), leaf stalk length (3.23 cm), leaf shape index (8.75) and LWR (9.29); HN provenance had the highest maximum leaf width (3.54 cm), leaf thickness (0.13 mm) and WWR (1.18). BS provenance had the highest 1/2 leaf width (3.24 cm). The coefficient of variation (c.v.) in the HN provenance was much higher than the other two provenances, ranging from 11.02% to 71.14%. The average coefficient of variation (c.v.) decreased in the following order: LWR (33.83%) > leaf shape index (31.46%) > leaf length (26.74%) > leaf stalk length (23.71%) > 1/2 leaf length (20.94%) > leaf thickness (18.09%) > maximum leaf width (18.07%) > WWR (6.81%) (Table 2 and Table S2). So the leaf shape and stalks between different provenances had significant differences, but the maximum and 1/2 width of leaves showed little difference. Leaves of BB provenance had the longest leaves and leaf stalks, while the HN provenance had the widest and thickest leaves among the three provenances.

As for leaf functional traits between the three provenances, there were only highly significant differences in single-leaf dry weight, indicating that the dry mass accumulation status showed quite a big difference. Leaves of BB provenance had highest single-leaf area (183.76 cm^2^), single-leaf fresh weight (3.16 g), dry weight (0.84 g) and specific leaf weight (0.0047 g/cm^2^), while BS provenance had the highest moisture content (75.66%) and specific leaf area (233.13 cm^2^/g). The average coefficient of variation (c.v.) decreased in the following order: single-leaf fresh weight (18.26%) > single-leaf area (18.17%) > single-leaf dry weight (10.91%) > specific leaf weight (8.79%) > specific leaf area (8.44%) > leaf moisture content (2.81%). The coefficients of variation (c.v.) in BB provenance of most functional indices were much higher than the other two provenances, while BB provenance had the highest variation in leaf moisture content (3.15%). Specific leaf area and specific leaf weight had higher variation degrees than the other two provenances (9.75% and 10.87%) (Table 3 and Table S3). Overall, BB provenance had the biggest and heaviest leaves, and it also showed the best ability for dry mass accumulation (highest single-leaf dry weight and specific leaf weight), while leaves of BS provenance had the highest moisture content.

### 3.2. Variations in Leaf Chlorophyll and Sugar Contents

**Figure 2:**
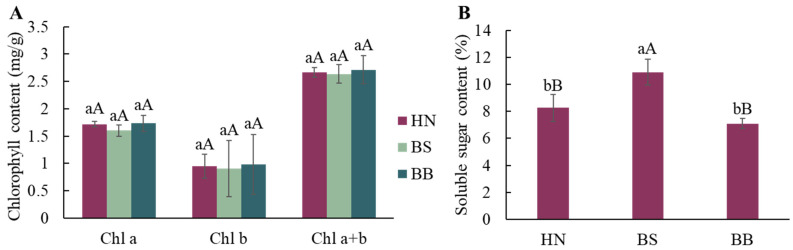
Leaf chlorophyll and soluble sugar contents between different *A. hainanensis* provenances (n = 3). Note: HN, BS and BB represented Hainan, Baise and Bobai provenances, respectively. The error bars represented the SD values of this index. Different uppercase letters indicated highly significant differences between different provenances of the same indices (*p* < 0.01), while different lowercase letters indicated significant differences between different provenances of the same indices (*p* < 0.05). (**A**) Leaf chlorophyll contents; (**B**) leaf soluble sugar content.

**Figure 3:**
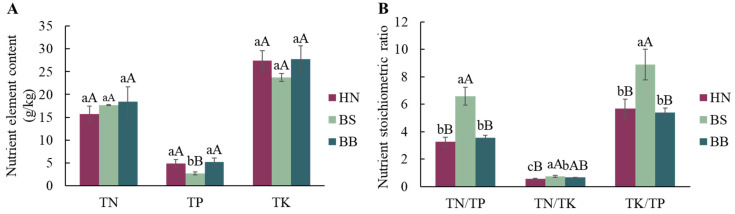
Nutrient contents (**A**) and stoichiometric ratios (**B**) in leaves of three *A. hainanensis* provenances (n = 3). Different uppercase letters indicated highly significant differences between different provenances of the same indices (*p* < 0.01), while different lowercase letters indicated significant differences between different provenances of the same indices (*p* < 0.05).

There were highly significant differences in soluble sugar content between the three *A. hainanensis* provenances, while chlorophyll contents had no significant differences (Figure 2A,B and Table S4). Leaves of BB provenance had the highest chlorophyll a, chlorophyll b and chlorophyll a + b contents (1.73 mg/g, 0.98 mg/g and 2.71 mg/g), while leaves of BS had the highest soluble sugar content (10.91%) (Figure 2A,B). In summary, BB and BS provenance had the highest chlorophyll and sugar contents, respectively, and the performance of HN provenance leaves was at the medium level. As for leaf nutrient contents, there were highly significant differences in TP content and nutrient stoichiometric ratios between the three *A. hainanensis* provenances. Leaves of BB provenance had the highest TN, TP and TK contents (18.40 g/kg, 5.18 g/kg and 27.80 g/kg), and the nutrient absorption status of HN provenance seedlings were better than that of BS provenance (Figure 3A and Table S5). For nutrient stoichiometric ratios, TN/TP, TN/TK and TK/TP all reached the maximum in BS provenance (6.59, 0.75 and 8.90), and all three provenances may be prone to N limitation (N/P < 14) (Figure 3B). BB provenance seedlings had best nutrient absorption abilities, and N might play an important role during the growth of *A. hainanensis* provenance seedlings.

### 3.3. Variations in Total Phytochemical Contents

**Figure 4:**
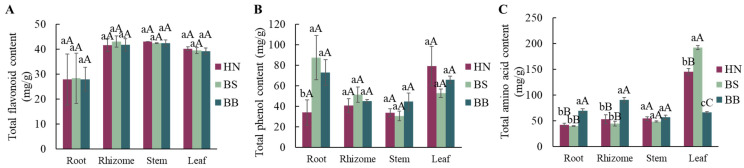
Chemical component contents in different organs of *A. hainanensis* provenances. (**A**) Total flavonoid content; (**B**) total phenol content; (**C**) total amino acid content. (n = 3). Different uppercase letters indicated highly significant differences between different provenances of the same indices (*p* < 0.01), while different lowercase letters indicated significant differences between different provenances of the same indices (*p* < 0.05).

There were no significant differences in total flavonoid contents among the four organs (roots, rhizomes, stems and leaves) between the three provenances, and for total phenol content, only roots showed significant differences among the three provenances. As for total amino acid, contents in roots, rhizomes and leaves all showed highly significant differences (except for stems) (Figure 4A–C and Table S6). BS provenance seedlings had highest total flavonoid content in its underground parts (28.34 mg/g in roots and 43.10 mg/g in rhizomes), while BB and HN provenances had the highest contents in stems (42.45 mg/g) and leaves (40.27 mg/g). Stem flavonoid contents in HN and BB provenances were higher than the other organs, while BS provenance had higher content in rhizomes (Figure 4A). As for total phenol content, it showed a similar trend to total flavonoids (87.71 mg/g and 51.49 mg/g in roots and rhizomes of BS provenance, 44.70 mg/g in stems of BB provenance and 79.21 mg/g in HN provenance leaves). BB and BS provenances had the highest contents in roots, while total phenol in HN provenance was dominant in leaves (Figure 4B). For total amino acid contents, roots, rhizomes and stems of BB provenance had the maxima rather than the other two provenances (69.02 mg/g, 90.36 mg/g and 56.51 mg/g), while BS provenance leaf content was significantly higher than the other two provenances (191.94 mg/g). Leaves were dominant organs for amino acid accumulation in both HN and BS provenances, while rhizomes were dominant in BB provenance (Figure 4C). In conclusion, aboveground and underground stems were the main accumulation organs for total flavonoid, roots and leaves accumulated more phenol, and leaves and rhizomes accumulated more amino acids. Different provenance seedlings should be chosen and cultivated with appropriate methods based on their characteristics in terms of the accumulation of various phytochemical contents, in order to better utilize their biological resources.

### 3.4. Correlation and Principal Component Analysis

(1) Correlation analysis

**Figure 5:**
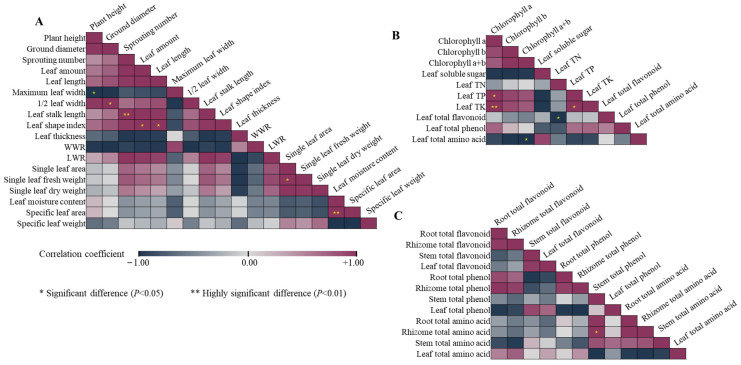
Correlation analysis between different indices of *A. hainanensis* provenances (note: (**A**) correlations between leaf morphological and functional characteristics; (**B**) correlations between leaf physiological indices and nutrients/total phytochemical contents; (**C**) correlations of total phytochemical contents between different organs. Indices in (**B**,**C**) represent the contents of these components including chlorophyll, sugar, nutrients, and total phytochemical contents).

There were different relationships between plant aboveground growth, leaf morphological traits, nutrient contents and chemical component contents among the three *A. hainanensis* provenances (Figure 5A–C). There were close relationships between plant height and stem diameter at the ground level and leaf/sprouting amount, indicating that the elongation, thickening of the aboveground part and the growth of leaves are interrelated. As for leaf morphological traits, leaf shape index (LSI) showed a positive correlation with leaf length, indicating that LSI would increase with increasing leaf length (demonstrating the trend of leaf growth). In addition, there were also (highly) significant correlations between leaf area and fresh/dry weight. Leaf moisture content also showed positive correlations with specific leaf area, suggesting that leaf weight would increase with increasing leaf areas, and leaf moisture content was also correlated with biomass accumulation (Figure 5A). For the relationships of physiological indices and nutrient/total phytochemical contents, there were positive relationships between chlorophyll a content and TN, TP, TK contents, and P and K might have a synergistic absorption effect (due to the significant positive correlation between them), indicating that nutrient status would relate to chlorophyll and even photosynthesis properties. N also showed negative correlations with total phytochemical contents, while P and K showed negative correlations with total flavonoid and amino acid contents, which was consistent with some plants (Figure 5B). For the total phytochemical contents between different organs, underground organs (roots, rhizomes) and the aboveground organs (stems, leaves) of *A. hainanensis* seedlings had their own respective synergistic effects, respectively, and there was also a significant positive correlation between stem total phenol and rhizome total amino acid contents (Figure 5C). In summary, growth and leaf morphological traits were tightly correlated with biomass accumulation. There were also negative correlations between N, P, K and most of the phytochemical contents, suggesting a probable trade-off between nutrient absorption and secondary metabolism.

(2) PCA

According to the replicates of all the indices, six principal components could be extracted separately from all the indices of the three provenances, and the cumulative contribution rates can all reach 95.183% (which is higher than 90%) (Table 4). PC1 showed the highest loadings on leaf TP/TK contents, total amino acid content in the root and rhizome, and total phenol content in the stem, indicating that this PC could represent phosphorus and potassium accumulation in leaves and amino acid accumulations in stems/rhizomes. The highest loadings of PC2 are mainly from leaf shape index, leaf length and leaf stalk length, which could represent the extent of leaf length. The loadings of leaf number, stem diameter at the ground level, plant height and width of leaves in PC3 were dominant, indicating that aboveground growth and leaf width could be represented. Sprouting number, leaf moisture content, single-leaf fresh weight (area) and specific leaf area had the highest loadings in PC4, indicating that the sprouting conditions, leaf moisture and biomass accumulation were dominant in PC4. PC5 had the highest loadings of leaf nitrogen/chlorophyll contents and phenol contents in roots and stems, and PC6 had the highest loadings of total flavonoid contents in roots and rhizomes (Table S7). So these PCs could mostly represent the leaf morphological characteristics, aboveground growth status, and nutrient and phytochemical contents. After calculating the comprehensive PC scores, we found that the comprehensive performances of BB provenance seedlings were the best in the three provenances, following by BS and HN. BS provenance performed better in plant growth and leaf morphological/functional indices, while HN provenance seedlings had better leaf nutrient and total phytochemical contents than BS (Table 5). Therefore, seedlings of BB provenance could be promoted and cultivated under the mixed valuable-tree-species forest in Nanning. Seedlings of HN provenance had better conditions of nutrients and phytochemical contents than the BS provenance seedlings, and seedlings of BS provenance had better growth conditions, but the medicinal quality needs to be improved.

## 4. Discussion

### 4.1. Morphological and Growth Traits Distinguish A. hainanensis Provenances

Leaf morphology and aboveground growth traits effectively distinguished the three *A. hainanensis* provenances. In our study, BB and BS provenances (from Guangxi) showed the characteristics of taller seedlings, bigger leaves, thicker stems and better biomass accumulation status than HN provenance, which probably relates to better adaptation to local understory conditions. Similarly, the morphological properties would be changed by ecological or other factors of the cultivation sites. As for leaf morphology, the shape would become much narrower and longer with the increase in latitude and altitude, which was consistent with *Erythropalum scandens* [32], *Michelia yunnanensis* [33] and many other medicinal plants. The original habitat of the HN provenance seedlings has a generally higher temperature compared to that of Nanning. The seedlings may encounter extreme low temperatures during winter after introduction to Nanning (which could affect the sprouting and growth of their leaves). In addition, the coefficient of variation in some morphological properties (such as sprouting numbers and leaf numbers) were higher than those of functional traits. Some research also showed that there were significant differences in morphological and functional traits between different parts and branches of plants [34]. In addition, we also found that there were no significant differences in some functional traits between the three provenances (like single-leaf area and moisture content), indicating that the adaptability of water utilization and leaf expansions were similar. Actually, the water usage strategies are changed when the introduction sites are quite far apart, causing different soil–leaf hydraulic conductivity and photosynthesis characteristics in some specific seasons or places [35]. Some anatomical structure indices show plasticity possibilities between different provenances, which needs further research [36]. Hainan is relatively close to Guangxi. Apart from possibly having some extremely cold environments, the remaining climate conditions do not differ significantly. This might be the reason why there are no significant differences among most functional trait germplasm sources. In conclusion, although the morphological characteristics of the HN provenance differed from two Guangxi provenances, most of the leaf functional traits did not show significant differences from BB and BS provenances. However, some seedlings with better growth performances could be selected for large-scale propagation.

### 4.2. Physiological and Phytochemical Contents Vary Among Provenances

The differences in photosynthesis pigments and osmotic adjustment substances between different provenances could cause different adaptation for environmental factors [37]. In this study, BB and BS provenances had the highest chlorophyll and sugar contents, indicating that they could better adapt to the understory environment. Plants adjusted their photosynthetic pigments contents to adapt to the external environment, and the differences in photosynthetic pigments contents among different varieties indicated that they also have different resistances to environmental factors [38,39]. There are significant differences in the content of osmotic regulatory substances among different provenances. Even after the environmental factors of the native habitat and the introduced habitat of the plant species change, they may still affect the content of osmotic regulatory substances in the plants [40]. BB and BS provenances have relatively strong adaptability to the forest-undergrowth environment in the region possibly due to originating from Guangxi. Although HN showed moderate growth, it accumulated higher flavonoid and phenol contents in leaves, suggesting a trade-off between growth and secondary metabolism, causing the differences in phytochemical contents.

Nutrient stoichiometric ratios could reflect the categories and degrees of nutrient limitation, which also provided certain guidance for nutrient additions during the subsequent cultivation and management process [41]. Our study showed that the ratios of N/P among the three *A. hainanensis* provenances were all lower than 14, which might be prone to N limitation; this situation is similar in many understory medicinal species. For example, the N/P ratio of *A. galanga* under *Dalbergia odorifera* forest was also lower than 14 (indicating probable slight N limitation), which might be correlated with a strong demand for N for both of their growth and development [28]. N, P and other nutrient limitations were common in plants inhabited in acid soil-based agroforestry systems in southern China, because an acid soil environment would affect nutrient release and absorption during developmental process [42]. The medicinal quality would correlate to nutrient absorption, environmental adaptability and many other aspects. In this study, there was a negative correlation between N and flavonoid content, and the accumulation levels of flavonoid, phenol and amino acid were all showing different trends among all the organs and flavonoids. For example, flavonoids always accumulated in leaves (even some tender-specific organs), and phenol content reached the maximum in plant roots or underground parts (such as *Ormosia henryi* and so on) [43], which was consistent with our study. As for the relationships between nutrient and total phytochemical contents, some research also showed that low-nitrogen treatments could enhance hydroxycinnamoyl esters/flavonoid concentrations (especially in roots) [44]. Amino acids always accumulated most in leaves, and the content also showed closely relationships with low nitrogen additions [45]. The negative correlation between N and flavonoid content supports this interpretation. Therefore, the physiological conditions of the plants usually performed better when the native habitats of the provenances were closed to the introduced areas. The negative correlation between N and flavonoid content supports a trade-off between primary and secondary metabolism in *A. hainanensis* in understory conditions.

### 4.3. PCA Integrates Multi-Trait Performance for Provenance Selection

PCA across different traits revealed that BB provenance had best comprehensive performances in both leaf and physiological/chemical aspects, making it suitable for mixed precious forest understory cultivation. So, there were close relationships between the original habitats and introduction sites. For example, SY and YH provenances of *Polygonatum cyrtonema* showed better performances in the three test sites of Zhejiang, China (with superior growth and medicinal conditions) [11]. But some other factors (like ecological adaptability, cultivation methods, etc.) could also influence the growth conditions of different provenances. Research showed that external provenances of *Casuarina junghuhniana* showed better growth performances than the local ones, and the enhancements of N-enrichment and foliage addition could also exhibit inter-specific and intra-specific variation [46]. The combination effects of shade and provenance differences were the main influence factors of growth status and terpenoid contents [9]. So, it is necessary to balance the internal and external effects of provenance introduction. As for correlation analysis, the results showed that there was a synergistic effect between the emergence of the aboveground parts (especially sprout and leaf amount). Moreover, the content of TP and TK in leaves was significantly positively correlated with the chlorophyll content, while total flavonoids had a significantly negative correlation with TN contents, which is consistent with many studies. Although N played a significant role in flavonoid biosynthesis as the main ingredient of precursor substances, excessive fertilization or N addition would decrease flavonoid content, and N also showed negative correlations in some specific organs or after some treatments of plants [47,48]. In conclusion, the physiological status of *A. hainanensis* seedlings was closely related to the effects of introduction, and N showed strong correlations with *A. hainanensis* flavonoids. Further studies on nutrient addition and wider introduction adaptability and cultivation of understory *A. hainanensis* provenances should be verified.

### 4.4. Summary and Prospect of A. hainanensis Provenance Selection and Cultivation

Based on the results of physiological and nutrient indices, BB provenance was recommended for cultivation under mixed precious forest. The additions of N, P, K and other nutrients might release the nutrient limitations, promote photosynthesis pigment synthesis and improve the growth status of various medicinal plants. As for different *A. hainanensis* provenance seedlings, appropriate N addition and other nutrients could enhance their growth status and total phytochemical contents. For example, the treatment ratio of 100:75:75 kg/ha N, P and K fertilization could promote yield and soil nutrient contents combined with proper B, Zn and Fe addition [49]. Dominant N addition in the compound manure–inorganic fertilizer would cause higher yield and quality of *Zingiber officinale* under *Toona ciliata* forest [50]. In addition, *Curcuma longa* could achieve better growth conditions and soil properties under 75:25 N fertilizer and farmyard manure treatment [51]. Proper addition of P and K could also improve the chlorophyll content and improve the fruit production rate, which could improve the medicinal organ yield of *A. hainanensis*. The HN and BS provenances may provide useful genetic material for breeding varieties characterized by high concentrations of chemical constituents in stems and leaves. Therefore, it is necessary to enhance the growth and development of some non-medicinal parts and utilize the biological resources of specific *A. hainanensis* provenances in future research. In addition, more provenances from the south and southwest/southeast of China (like Yunnan, Guangdong and other provinces) could be introduced from their original habitats to the understory space of Guangxi, in order to find their tolerance for canopy environments.

## 5. Conclusions

In conclusion, there were significant differences in growth, physiological characteristics and phytochemical contents among the three *A. hainanensis* provenances (two provenances from Guangxi performed better than that from Hainan). For agronomic performance, BB provenance showed better performances while BS provenance had thicker stems. According to the results of nutrient and total phytochemical comprehensive analysis, BB and HN provenances performed better than BS, while BB had better growth status than HN (including larger leaves, higher nutrient accumulation and some phytochemical contents in specific organs). A negative correlation between N and flavonoid content suggested a trade-off between primary and secondary metabolism. It is also necessary to enhance the cultivation and fertilization methods for *A. hainanensis* cultivation in various understory space, and the analysis of the genetic diversity among different provenances should also be strengthened. These findings provide a scientific basis for provenance selection in subtropical agroforestry systems.

## Figures and Tables

**Table 1:** Growth performances of different *Alpinia hainanensis* provenances (n = 30).

Provenance	Plant Height (cm)	Stem Diameter at the Ground Level (mm)	Sprouting Number	Leaf Number
HN	42.72 ± 6.47 bB (15.15%)	3.87 ± 0.67 bB (17.31%)	2.03 ± 0.32 bA (15.76%)	10.10 ± 2.77 cC (27.43%)
BS	73.79 ± 20.99 aA (28.45%)	6.63 ± 1.73 aA (26.09%)	2.33 ± 1.12 abA (48.07%)	14.87 ± 6.18 bB (41.56%)
BB	66.69 ± 13.83 aA (20.74%)	6.37 ± 1.51 aA (23.70%)	2.67 ± 0.96 aA (35.96%)	18.57 ± 6.26 aA (33.71%)
Average c.v.	21.47%	22.37%	33.26%	34.23%

Note: The data in the table represent “Mean ± SD (Standardize Deviation)”. Different uppercase letters in the same column indicate that the traits of different provenances have highly significant differences (*p* < 0.01), while different lowercase letters in the same column indicate that the traits of different varieties have significant differences (*p* < 0.05). The percentage in parentheses represents the coefficient of variation (c.v.%) of this index.

**Table 2:** Leaf morphological traits of different *A. hainanensis* provenances (n = 30).

Provenance	Leaf Length (cm)	Maximum Leaf Width (cm)	1/2 Leaf Width (cm)	Leaf Stalk Length (cm)
HN	20.04 ± 9.89 bB (49.35%)	3.54 ± 0.71 aA (20.06%)	3.08 ± 0.84 aA (27.27%)	1.54 ± 0.53 cC (34.42%)
BS	25.94 ± 4.57 aA (17.62%)	3.43 ± 0.58 aA (16.91%)	3.24 ± 0.59 aA (18.21%)	2.33 ± 0.48 bB (20.60%)
BB	29.27 ± 3.88 aA (13.26%)	3.46 ± 0.59 aA (17.05%)	3.23 ± 0.56 aA (17.34%)	3.23 ± 0.52 aA (16.10%)
Average c.v.	26.74%	18.07%	20.94%	23.71%
Provenance	Leaf shape index	Leaf thickness (mm)	WWR	LWR
HN	5.90 ± 3.87 bB (65.59%)	0.13 ± 0.02 aA (15.38%)	1.18 ± 0.13 aA (11.02%)	7.52 ± 5.35 aA (71.14%)
BS	7.67 ± 0.92 aA (11.99%)	0.12 ± 0.02 aA (16.67%)	1.07 ± 0.03 bB (2.80%)	8.18 ± 1.02 aA (12.47%)
BB	8.75 ± 1.47 aA (16.80%)	0.09 ± 0.02 bB (22.22%)	1.06 ± 0.07 bB (6.60%)	9.29 ± 1.66 aA (17.87%)
Average c.v.	31.46%	18.09%	6.81%	33.83%

Note: The data in the table represent “Mean ± SD (Standardize Deviation)”. Different uppercase letters in the same column indicate that the traits of different provenances have highly significant differences (*p* < 0.01), while different lowercase letters in the same column indicate that the traits of different varieties have significant differences (*p* < 0.05). The percentage in parentheses represents the coefficient of variation (c.v.%) of this index.

**Table 3:** Leaf area and some functional traits of different *A. hainanensis* provenances (n = 3).

Provenance	Single-Leaf Area (cm^2^)	Single-Leaf Fresh Weight (g)	Single-Leaf Dry Weight (g)
HN	131.15 ± 21.43 aA (16.34%)	2.25 ± 0.29 aA (12.89%)	0.60 ± 0.04 bB (6.67%)
BS	126.36 ± 22.97 aA (18.18%)	2.25 ± 0.48 aA (21.33%)	0.54 ± 0.07 bB (12.96%)
BB	183.76 ± 36.73 aA (19.99%)	3.16 ± 0.65 aA (20.57%)	0.84 ± 0.11 aA (13.10%)
Average c.v.	18.17%	18.26%	10.91%
Provenance	Moisture content (%)	Specific leaf area (cm^2^/g)	Specific leaf weight (g/cm^2^)
HN	73.34 ± 1.64 aA (2.24%)	218.50 ± 21.31 aA (9.75%)	0.0046 ± 0.0005 aA (10.87%)
BS	75.66 ± 2.30 aA (3.04%)	233.13 ± 18.46 aA (7.92%)	0.0043 ± 0.0003 aA (6.98%)
BB	72.93 ± 2.30 aA (3.15%)	216.18 ± 16.54 aA (7.65%)	0.0047 ± 0.0004 aA (8.51%)
Average c.v.	2.81%	8.44%	8.79%

Note: The data in the table represent “Mean ± SD (Standardize Deviation)”. Different uppercase letters in the same column indicate that the traits of different provenances have highly significant differences (*p* < 0.01), while different lowercase letters in the same column indicate that the traits of different varieties have significant differences (*p* < 0.05). The percentage in parentheses represents the coefficient of variation (c.v.%) of this index.

**Table 4:** Principal component extraction of all the indices for different *A. hainanensis* provenances.

PC	Initial Eigenvalue			Extract the Sum of Squared Loads		
	Total	Variance Percentage	Accumulation %	Total	Variance Percentage	Accumulation %
1	9.933	26.846	26.846	9.933	26.846	26.846
2	8.971	24.246	51.092	8.971	24.246	51.092
3	6.858	18.535	69.626	6.858	18.535	69.626
4	4.024	10.875	80.502	4.024	10.875	80.502
5	2.967	8.019	88.521	2.967	8.019	88.521
6	2.465	6.662	95.183	2.465	6.662	95.183

**Table 5:** Principal component score for different *A. hainanensis* provenances.

Provenance	PC Scores						Comprehensive Score	Order
	PC1	PC2	PC3	PC4	PC5	PC6		
HN	0.051	−3.201	−1.156	0.656	−0.424	0.370	−0.960	3
BS	−3.526	1.192	0.658	−0.530	0.644	−0.110	−0.577	2
BB	3.476	2.010	0.498	−0.126	−0.220	−0.260	1.536	1

## Data Availability

All relevant data are within the manuscript and its additional files.

## Supplementary Materials

The following supporting information can be downloaded at: https://www.mdpi.com/article/10.3390/plants15111602/s1, Table S1: Analysis of variance table for aboveground growth indices of different *A. hainanensis* provenances; Table S2: Analysis of variance table for leaf morphological indices of different *A. hainanensis* provenances; Table S3: Analysis of variance table for leaf functional indices of different *A. hainanensis* provenances; Table S4: Analysis of variance table for leaf chlorophyll and soluble sugar contents of different *A. hainanensis* provenances; Table S5: Analysis of variance table for leaf nutrient contents and nutrient stoichiometric ratios of different *A. hainanensis* provenances; Table S6: Analysis of variance table for total phytochemical contents among all the organs of different *A. hainanensis* provenances; Table S7 Factor loading table of all the indices for different *A. hainanensis* provenances.
