# Supplementary material for: Provenance Variation in Morphology, Nutrient Status and Total Phytochemical Contents of Alpinia hainanensis K. Schum. Under a Mixed Valuable-Tree-Species Forest
Source: Plants (Basel). 2026 May 23;15(11):1602. doi: 10.3390/plants15111602 (PMC13258854; doi:10.3390/plants15111602)
Supplement: Supplementary file 1 [file plants-15-01602-s001.zip › plants-4302029-supplementary.pdf]

Supplementary materials

Table S1. Analysis of variance table for aboveground growth indices of different *A. hainanensis* provenances

Index	Source	SS	df	MS	*F*-value	*P*-Value
Plant height	Between Group	15909.225	2	7954.613	35.425	0
	Within Group	19535.921	87	224.551		
	Total	35445.146	89			
Stem diameter at the ground level	Between Group	139.802	2	69.901	36.572	0
	Within Group	166.286	87	1.911		
	Total	306.088	89			
Sprouting number	Between Group	6.022	2	3.011	3.951	0.023
	Within Group	66.3	87	0.762		
	Total	72.322	89			
Leaf number	Between Group	1080.956	2	540.478	19.056	0
	Within Group	2467.533	87	28.362		
	Total	3548.489	89			

Table S2. Analysis of variance table for leaf morphological indices of different *A. hainanensis* provenances

Index	Source	SS	df	MS	*F*-value	*P*-Value
	Between Group	1312.412	2	656.206	14.715	0
Leaf length	Within Group	3879.589	87	44.593		
	Total	5192.001	89			
	Between Group	0.174	2	0.087	0.222	0.801
Maximum leaf width	Within Group	34.115	87	0.392		
	Total	34.289	89			
	Between Group	6.481	2	3.241	1.15	0.321
1/2 leaf width	Within Group	245.11	87	2.817		
	Total	251.591	89			
	Between Group	42.575	2	21.288	82.319	0
Leaf stalk length	Within Group	22.498	87	0.259		
	Total	65.074	89			
	Between Group	124.406	2	62.203	10.353	0
Leaf shape index	Within Group	522.722	87	6.008		
	Total	647.128	89			
	Between Group	0.027	2	0.013	28.124	0
Leaf thickness	Within Group	0.041	87	0		
	Total	0.068	89			
	Between Group	0.274	2	0.137	17.753	0
WWR	Within Group	0.671	87	0.008		
	Total	0.945	89			
	Between Group	48.135	2	24.068	2.228	0.114
LWR	Within Group	939.859	87	10.803		
	Total	987.994	89			

Table S3. Analysis of variance table for leaf functional indices of different *A. hainanensis* provenances

Index	Source	SS	df	MS	*F*-value	*P*-Value
Single leaf area	Between Group	6085.516	2	3042.758	3.908	0.082
	Within Group	4671.082	6	778.514		
	Total	10756.598	8			
Single leaf fresh weight	Between Group	1.65	2	0.825	3.38	0.104
	Within Group	1.464	6	0.244		
	Total	3.115	8			
Single leaf dry weight	Between Group	0.155	2	0.077	12.531	0.007
	Within Group	0.037	6	0.006		
	Total	0.192	8			
Moisture content	Between Group	12.957	2	6.479	1.465	0.303
	Within Group	26.54	6	4.423		
	Total	39.497	8			
Specific leaf area	Between Group	506.722	2	253.361	0.711	0.528
	Within Group	2137.179	6	356.197		
	Total	2643.901	8			
Specific leaf weight	Between Group	0	2	0	0.793	0.495
	Within Group	0	6	0		
	Total	0	8			

Table S4. Analysis of variance table for leaf chlorophyll and soluble sugar contents of different *A. hainanensis* provenances

Index	Source	SS	df	MS	*F*-value	*P*-Value
Chlorophyll a content	Between Group	0.00	2	0	0.04	0.961
	Within Group	0.02	6	0.003		
	Total	0.02	8			
Chlorophyll b content	Between Group	0.009	2	0.004	0.043	0.958
	Within Group	0.610	6	0.102		
	Total	0.619	8			
Chlorophyll a+b content	Between Group	0.010	2	0.005	0.038	0.963
	Within Group	0.772	6	0.129		
	Total	0.782	8			
Soluble sugar content	Between Group	22.884	2	11.442	16.727	0.004
	Within Group	4.104	6	0.684		
	Total	26.988	8			

Table S5. Analysis of variance table for leaf nutrient contents and nutrient stoichiometric ratios of different *A. hainanensis* provenances

Index	Source	SS	df	MS	*F*-value	*P*-Value
Total nitrogen content	Between Group	11.454	2	5.727	1.978	0.219
	Within Group	17.371	6	2.895		
	Total	28.826	8			
Total phosphorus content	Between Group	10.836	2	5.418	18.678	0.003
	Within Group	1.74	6	0.29		
	Total	12.576	8			
Total potassium content	Between Group	29.935	2	14.967	2.268	0.185
	Within Group	39.591	6	6.598		
	Total	69.525	8			
TN/TP	Between Group	20.366	2	10.183	55.446	0
	Within Group	1.102	6	0.184		
	Total	21.468	8			
TN/TK	Between Group	0.043	2	0.022	16.395	0.004
	Within Group	0.008	6	0.001		
	Total	0.051	8			
TK/TP	Between Group	22.677	2	11.339	18.792	0.003
	Within Group	3.62	6	0.603		
	Total	26.297	8			

Table S6. Analysis of variance table for total phytochemical contents among all the organs of different *A. hainanensis* provenances

Index	Source	SS	df	MS	*F*-value	*P*-Value
Root total flavonoid content	Between Group	0.366	2	0.183	0.002	0.998
	Within Group	456.616	6	76.103		
	Total	456.981	8			
Root total phenol content	Between Group	4624.753	2	2312.377	8.861	0.016
	Within Group	1565.845	6	260.974		
	Total	6190.598	8			
Root total amino acid content	Between Group	1618.663	2	809.332	95.301	0
	Within Group	50.954	6	8.492		
	Total	1669.618	8			
Rhizome total flavonoid content	Between Group	3.878	2	1.939	0.325	0.734
	Within Group	35.755	6	5.959		
	Total	39.634	8			
Rhizome total phenol content	Between Group	176.904	2	88.452	2.618	0.152
	Within Group	202.71	6	33.785		
	Total	379.614	8			
Rhizome total amino acid content	Between Group	3615.59	2	1807.795	46.013	0
	Within Group	235.734	6	39.289		
	Total	3851.324	8			
Stem total flavonoid content	Between Group	0.936	2	0.468	0.84	0.477
	Within Group	3.34	6	0.557		
	Total	4.276	8			
Stem total phenol content	Between Group	327.399	2	163.7	4.483	0.064
	Within Group	219.074	6	36.512		
	Total	546.473	8			
Stem total amino acid content	Between Group	103.213	2	51.607	4.834	0.056
	Within Group	64.049	6	10.675		
	Total	167.263	8			
Leaf total flavonoid content	Between Group	1.759	2	0.88	0.683	0.54
	Within Group	7.729	6	1.288		
	Total	9.488	8			
Leaf total phenol content	Between Group	1046.797	2	523.398	3.906	0.082
	Within Group	803.987	6	133.998		
	Total	1850.784	8			
Leaf total amino acid content	Between Group	24352.699	2	12176.349	638.273	0
	Within Group	114.462	6	19.077		
	Total	24467.161	8			

Table S7. Factor loading table of all the indices for different *A. hainanensis* provenances

Indices	PC1	PC2	PC3	PC4	PC5	PC6
Plant height	-0.311	0.625	0.609	-0.302	-0.193	-0.049
Stem diameter at the ground level	-0.115	0.372	0.776	-0.378	-0.121	0.257
Sprouting number	-0.363	0.191	0.443	0.760	-0.066	0.097
Leaf amount	-0.006	0.136	0.895	-0.045	-0.147	0.384
Leaf length	0.354	0.752	0.372	-0.337	0.175	-0.107
Maximum leaf width	0.433	-0.520	0.556	0.420	0.066	0.133
1/2 leaf width	0.409	-0.492	0.627	0.383	0.135	0.118
Leaf stalk length	0.506	0.762	0.223	-0.151	-0.138	-0.196
Leaf shape index	-0.013	0.822	0.081	-0.398	0.293	-0.139
Leaf thickness	-0.316	-0.676	0.550	0.260	0.263	0.052
WWR	-0.158	-0.014	-0.831	-0.029	-0.507	0.080
LWR	-0.087	0.827	-0.275	-0.423	0.043	-0.117
Single leaf area	0.552	0.603	-0.193	0.495	-0.151	-0.066
Single leaf fresh weight	0.501	0.627	-0.112	0.533	-0.149	-0.038
Single leaf dry weight	0.751	0.488	-0.055	0.357	-0.192	-0.025
Leaf moisture content	-0.648	0.481	-0.216	0.543	0.080	-0.013
Specific leaf area	-0.523	0.475	-0.449	0.449	0.062	-0.200
Specific leaf weight	0.520	-0.492	0.419	-0.466	-0.107	0.122
Chlorophyll a content	0.496	-0.138	-0.564	0.039	0.349	-0.493
Chlorophyll b content	0.286	-0.154	-0.427	-0.518	0.274	0.395
Chlorophyll a+b content	0.272	-0.091	-0.504	-0.473	0.325	0.393
Leaf soluble sugar content	-0.944	0.150	-0.008	-0.053	0.051	-0.191
Leaf TN content	0.302	0.497	0.255	-0.111	0.717	0.087
Leaf TP content	0.898	-0.162	-0.238	0.039	0.024	0.170
Leaf TK content	0.659	-0.080	0.107	0.309	0.249	0.464
Root total flavonoid content	-0.114	0.382	-0.441	-0.248	-0.356	0.658
Root total phenol content	-0.239	0.721	0.364	0.069	0.414	-0.259
Root total amino acid content	0.872	0.447	0.116	0.016	0.013	-0.073
Rhizome total flavonoid content	-0.371	0.604	-0.339	0.284	-0.007	0.534
Rhizome total phenol content	-0.426	0.382	0.766	0.114	0.089	0.219
Rhizome total amino acid content	0.876	0.302	0.270	-0.008	-0.171	-0.113
Stem total flavonoid content	0.122	-0.192	-0.620	0.341	0.545	0.319
Stem total phenol content	0.831	0.226	-0.146	0.067	0.450	-0.079
Stem total amino acid content	0.617	-0.001	-0.026	0.023	-0.729	-0.032
Leaf total flavonoid content	-0.013	-0.792	0.156	-0.279	-0.059	-0.482
Leaf total phenol content	0.470	-0.791	0.115	0.090	0.047	-0.123
Leaf total amino acid content	-0.943	-0.239	0.008	-0.036	0.211	0.080
